# Comparative metabolite profiling of four polyphenol rich Morus leaves extracts in relation to their antibiofilm activity against *Enterococcus faecalis*

**DOI:** 10.1038/s41598-022-24382-4

**Published:** 2022-11-23

**Authors:** Mohamed A. Salem, Maha M. Salama, Shahira M. Ezzat, Yomna A. Hashem

**Affiliations:** 1 Department of Pharmacognosy and Natural Products, Faculty of Pharmacy, Menoufia University, Gamal Abd El Nasr St., Shibin Elkom, 32511 Menoufia Egypt; 2 Department of Pharmacognosy, Faculty of Pharmacy, Cairo University, Kasr El-Aini St, Cairo, 11562 Egypt; 3 Department of Pharmacognosy, Faculty of Pharmacy, The British University in Egypt, Suez Desert Road, El Sherouk City, Cairo, 11837 Egypt; 4 Department of Pharmacognosy, Faculty of Pharmacy, October University for Modern Sciences and Arts (MSA), Giza, 12451 Egypt; 5 Department of Microbiology, Faculty of Pharmacy, The British University in Egypt, Suez Desert Road, El Sherouk City, Cairo, 11837 Egypt

**Keywords:** Microbiology, Plant sciences, Health care

## Abstract

Enterococci are a common cause of urinary tract infections. The severity of enterococcal infections is associated with their ability to form biofilms. Morus leaves are known as a natural antibacterial, however, their antibiofilm activity against Enterococcus remains unveiled. This study aimed to evaluate the ability of four polyphenol-rich Morus leaves extracts (*Morus nigra*, *M*. *rubra*, *M*. *macroura,* and *M*. *alba)* to inhibit biofilm formed by enterococcal clinical isolates in relation to their metabolic profiling. Results revealed that 48% of the isolates formed strong biofilm, 28% formed moderate biofilm, 20% formed weak biofilm, and only 4% did not form a biofilm. The strong biofilm-forming isolates were *E. faecalis,* and hence were chosen for this study*.* The antibiofilm activity of the four polyphenol-rich Morus leaves extracts revealed that the *M*. *nigra* extract exhibited the highest percentage of biofilm inhibition followed by *M*. *rubra* then *M*. *macroura* and the least inhibition was detected in *M*. *alba,* and these results were in accordance with the phenolic and flavonoid contents of each extract*.* UPLC-ESI-MS/MS identified 61 polyphenolic compounds in the four extracts. Further, multivariate analysis confirmed clear segregation of *M. nigra* from the other species suggesting disparity in its metabolome, with accumulation of flavonoids, anthocyanidins, phenolic acids and coumarin derivatives. Quercetin and kaempferol glycosides were found to be positively and significantly correlated to the antibiofilm activity. In conclusion, *M. nigra* ethanolic extracts showed the highest phenolic content and antibiofilm activity and they could be developed as a complementary treatment for the development of antimicrobial agents.

## Introduction

Enterococci are Gram-positive, catalase-negative, facultative anaerobic organisms that can occur as single cocci and in chains^1^. Despite being commensal of the gastrointestinal tract of man and animals are believed to be harmless and used as probiotics^2^. Enterococci are emerging as one of the main nosocomial pathogens^3^ causing a wide variety of infections including urinary tract infections, endocarditis, surgical wound infections, and bacteremia^4^. Among several enterococcal species identified, the most common species associated with infections are *Enterococcus faecalis* and *Enterococcus faecium*^5^. The ability of enterococci to resist the action of many antibiotics used has played an important role in increasing the rate of prolonged enterococcal infections^6^. The antimicrobial resistance of enterococci can either be intrinsic or acquired via mobile resistance genes on plasmids and transposons^7^.

In addition to resistance, enterococci can adhere to different surfaces, forming a biofilm. Biofilms are communities of bacterial cells attached irreversibly to living or non-living surfaces and enclosed in an extracellular polymeric matrix of carbohydrates, protein, and DNA^8^. The extracellular matrix protects bacterial cells in the biofilm making them difficult to eradicate and leading to persistent infections^9^. *E. faecalis* in the biofilm tolerates higher concentrations of antibiotics than their planktonic counterparts^10^. The resistance to different antibiotics due to biofilm formation urges the finding of novel agents to treat infections^11^.

Plant secondary metabolites like polyphenols are reported to have antibacterial and antibiofilm activities^12^. Family Moraceae (Mulberry family or fig family) comprises about 38 genera and over 1100 species. They are mostly widespread in tropical and subtropical regions and less in temperate climates; however, their distribution is cosmopolitan overall. The fruits are edible with high nutritional value, and they is the food source for silkworm. Several *Morus* species have been identified, while the commonly used species are *M*. *alba* (white mulberry), *M*. *macroura* (king white mulberry, long mulberry), *M*. *rubra* (red mulberry) and *M*. *nigra* (black mulberry). *Morus* leaves have been reported to treat diabetes mellitus and prevent throat infections, irritations and inflammations^13^.

Several recent research have demonstrated the anti-inflammatory, antiviral, anti-hyperglycemic, cytotoxic, antihypertensive, and anti-hyperlipidemic effects of several Morus species due to the presence of polyphenols such as flavonoids and coumarin derivatives^14,15^. Since the leaves have been recommended for bacterial infection, this study was carried out to explore the capability of four polyphenol-rich Morus leaves extracts (*Morus nigra*, *M*. *rubra*, *M*. *macroura* and *M*. *alba*) to inhibit the biofilm formed by enterococcal clinical isolates which are the causative agent for urinary tract infections. Further, a non-targeted metabolomics approach was performed to annotate the metabolites enriched in the tested extracts. These investigations revealed that the maximum biofilm inhibition was observed with *M*. *nigra* leaves extract. Intriguingly, metabolite markers from polyphenols discriminated *M*. *nigra* leaves extract from other tested species.

## Materials and methods

### Preparation of different Morus leaves extracts

Morus leaves (*Morus nigra*, *M*. *rubra*, *M*. *macroura* and *M*. *alba*) were collected from the same farm located in Banha, Qalyubia Governorate (Egypt), in June 2019. The harvesting of the plant material was done by hand-picking technique. The leaves were then authenticated by the Agricultural Research Centre (ARC), 9 Cairo University Rd, Oula, Giza district, Giza (Egypt). The plant experiments were performed in accordance with relevant guidance and regulations. The leaves of the four Morus species were shade-dried for three days and eventually ground to a fine powder using an electric grinder. The powdered material was extracted by maceration in 70% ethanol for 72 h at ambient temperature^16^. The obtained extracts were filtered, and the filtrates were evaporated at a temperature, not exceeding 55 °C under reduced pressure in a rotary evaporator to dryness. The dried extracts were directly subjected to further analysis.

### Metabolite profiling by ultra-performance liquid chromatography- high resolution-electrospray ionization tandem mass spectrometry (UPLC-ESI–MS/MS)

The dried extracts (10 mg, each) were dissolved in 1 mL of HPLC-grade aqueous methanol (50%, v/v). Samples were quickly vortexed, sonicated for 5 min and finally centrifuged at 5000 g for 5 min at 4 °C. An injection volume of 2 µL was loaded on a reversed-phase column in a 20 min gradient described previously^17^. Samples were subjected to high resolution mass spectrometry analysis using electrospray ionization (ESI) in positive and negative ionization modes^18^.

### Determination of total phenolics and flavonoids contents

The total phenolic content in the leaves of the four investigated extracts was determined spectrophotometrically using a rapid microtiter plate Folin-Ciocalteu assay^19^. High-throughput microplate assays were applied for screening the flavonoid content^20^. Gallic acid and rutin were used as standard phenolic acid and flavonoid, respectively. Briefly, Morus extracts were prepared in concentrations of 3 mg/mL in methanol/water (9:1 v/v) and serial dilutions of standards were used at different concentrations (500–7.8 µg/mL for gallic acid). The average of six replicates was taken to produce a calibration curve (average *R*^2^ = 0.9986). The total phenolic concentration was expressed as mg gallic acid equivalent (GAE) g^−1^ of the dried sample. For rutin, the average of six replicates was taken to generate a calibration curve (average R^2^ = 0.9946 at a concentration range of 1000–50 µg/mL). The total flavonoid concentration was expressed as mg rutin equivalent (rutin) g^−1^ of the dried sample. The absorbance was measured at λ_max_ 630 and 510 nm for total phenolics and flavonoids contents, respectively. The results were recorded using a microplate reader (FluoStar® Omega).

### Bacterial isolates

Twenty-five isolates were collected from Egyptian clinical laboratories from patients with urinary tract infections (UTIs) from 2019 to 2020. The bacteria were identified to genus level by surface streaking on Enterococcosel agar (Difco Laboratories, USA) and chromogenic UTI agar (Conda, Spain) and staining of pure colonies by Gram stain. The identity of the isolates was confirmed by catalase and 6.5% NaCl tolerance tests.

### Identification of the isolates by PCR assay

Identification to species level was done by PCR with specific primers amplifying the *ddl* gene of *E. faecalis* and *E. faecium*. *E. faecalis* ATCC29212 and *E. faecium* ATCC700425 were used as reference strains. The DNA was extracted by boiling a few colonies in TRIS- EDTA buffer. Primers used for identification were 5'ATCAAGTACAGTTAGTCTTTA-3' and 5'-AACGATTCAAAGCTAACT-3' for *E. faecalis* and 5'-CCAAGGCTTCTTAGAGA-3' and 5'-CATCGTGTAAGCTAACTTC-3' for *E. faecium*^21^. Reaction mixtures were done in 0.2 mL reaction tubes, each with 25 µL reaction mixtures. The mixture consisted of 0.25 µg extracted DNA, 1.5 mM MgCl_2_, 10 pM of each primer, 200 µM of each deoxyribonucleotide, 5 X reaction buffer, and 0.5 U Taq polymerase (Qiagen, Germany) PCR amplification was performed in a SensoQuest (Germany) thermocycler. The amplification conditions were an initial denaturation step at 95 °C for 2 min, followed by 30 cycles of denaturation at 95 °C for 30 s, annealing at 95 °C for 30 s, and extension at 72 °C for 30 s. The reaction was concluded by a final extension step at 72 °C for 5 min. PCR products were analyzed by gel electrophoresis and visualized under UV light^22^.

### Biofilm assay

#### Congo-red agar biofilm assay

An overnight culture of the tested organisms was cultivated on Congo red agar plates (CRA). CRA plates were prepared by adding 0.8 g of Congo red dye (Fisher Scientific, USA) and 36 g of sucrose (Merck, Germany) to one liter of brain heart infusion agar (BHI agar, from Oxoid, UK). The plates were incubated for 24 h at 37 °C. A color scale was used to classify biofilm strength including red, almost black, black, and very black with crystalline colonies. Very black with crystalline colonies and black colonies were considered strong biofilm producer isolates, while almost black colors were indicative of a weak biofilm production activity and isolates with red colonies were classified as isolates unable to produce the biofilm^23,24^.

### Crystal violet assay

The strength of the biofilm formed was assessed by the Crystal Violet assay according to Christensen method with modification. An overnight culture of the tested organisms was inoculated in Trypticase Soy Broth (TSB) (Oxoid, UK), containing 0.5% glucose, and incubated at 37 °C for 24 h. After incubation, the culture density was adjusted to 0.5 McFarland by spectrophotometry (Unicam, UK). Cultures with adjusted concentrations were further diluted 100 times with TSB containing 0.5% glucose. Sterile flat-bottom 96 well microtiter plates were inoculated aseptically with 200 μL of the diluted cultures, and each isolate was added in triplicate. Negative control of TSB containing 0.5% glucose alone was also added, and the microtiter plates were incubated at 37 °C for 24 h. After overnight incubation, the contents of the plates were discarded, and the wells were washed with 200 μl saline three times and left to dry. Adherent cells were fixed with methanol and stained with 150 μl of 1% (W/V) Crystal Violet for 15 min; excess stain was removed by washing with tape water, and the plates were left to dry. The Crystal Violet bound to adherent cells was resolubilized by adding 200 μl 33% glacial acetic acid. The optical density (OD) was measured at wavelength 545 nm in a plate reader (Biotek, USA). The average of three optical density values was taken and the standard deviation was calculated^25–27^.

The strength of biofilm was classified according to the OD readings as follows:

O.D. < O.D.c (O.D. of the negative control) = non-adherent, O.D.c < O.D. < (2 × O.D.c) = weakly adherent, (2 × O.D.c) < O.D. < (4 × O.D.c) = moderately adherent and (4 × O.D.c) < O.D. = strongly adherent^28^.

### Antibiofilm assay of Morus leaves extract

The antibiofilm activity of Morus leaves extracts against *E. faecalis* clinical isolates was done using Crystal Violet assay. In 96 well microtiter plate flat bottom, 100 μL of TSB containing 0.5% glucose with serial dilutions of different mulberry extracts initiated with 250 mg/ml was added to the wells. Bacterial suspensions whose concentration was adjusted to 0.5 McFarland and diluted 100 times were also added to the wells. Positive controls including broth and tested isolates, and negative controls including broth alone were also included, and each isolate was done in triplicate. The plates were incubated at 37 °C for 24 h. The biofilm formed in the presence of morus extracts was determined by Crystal Violet assay as described above. The biofilm inhibition percentage was calculated using the following formula: [(OD growth control − OD sample)/OD growth control] × 100^29^.

### Statistical analysis

LC/MS data were processed using the ToxID 2.1.2 and Xcalibur 2.1 software package (Thermo Fisher Scientific Inc., USA). In order to perform multivariate analysis, all the obtained data were 1og_10_-transformed and scaled prior to analysis using SIMCA (version 14.1, Umetrics, Umeå, Sweden) and MetaboAnalyst 5.0^30^. The polyphenols abundance heat maps were generated using Multiple Experiment Viewer (MeV_4_9_0)^31^. All statistical analyses, including descriptive statistics and hypothesis tests (e.g., Chi-Square, Fisher Exact Test, ANOVA, and Student t-test were performed on Data Desk v. 6.3 (Data Description Inc., Ithaca, NY, USA) and GraphPad Prism (GraphPad Software Tools, Inc., La Jolla, CA, USA). *P* values less than 0.05 were considered significant.

## Results

### Identification of metabolites in the four polyphenol-rich Morus leaves extracts

**Figure 1:**
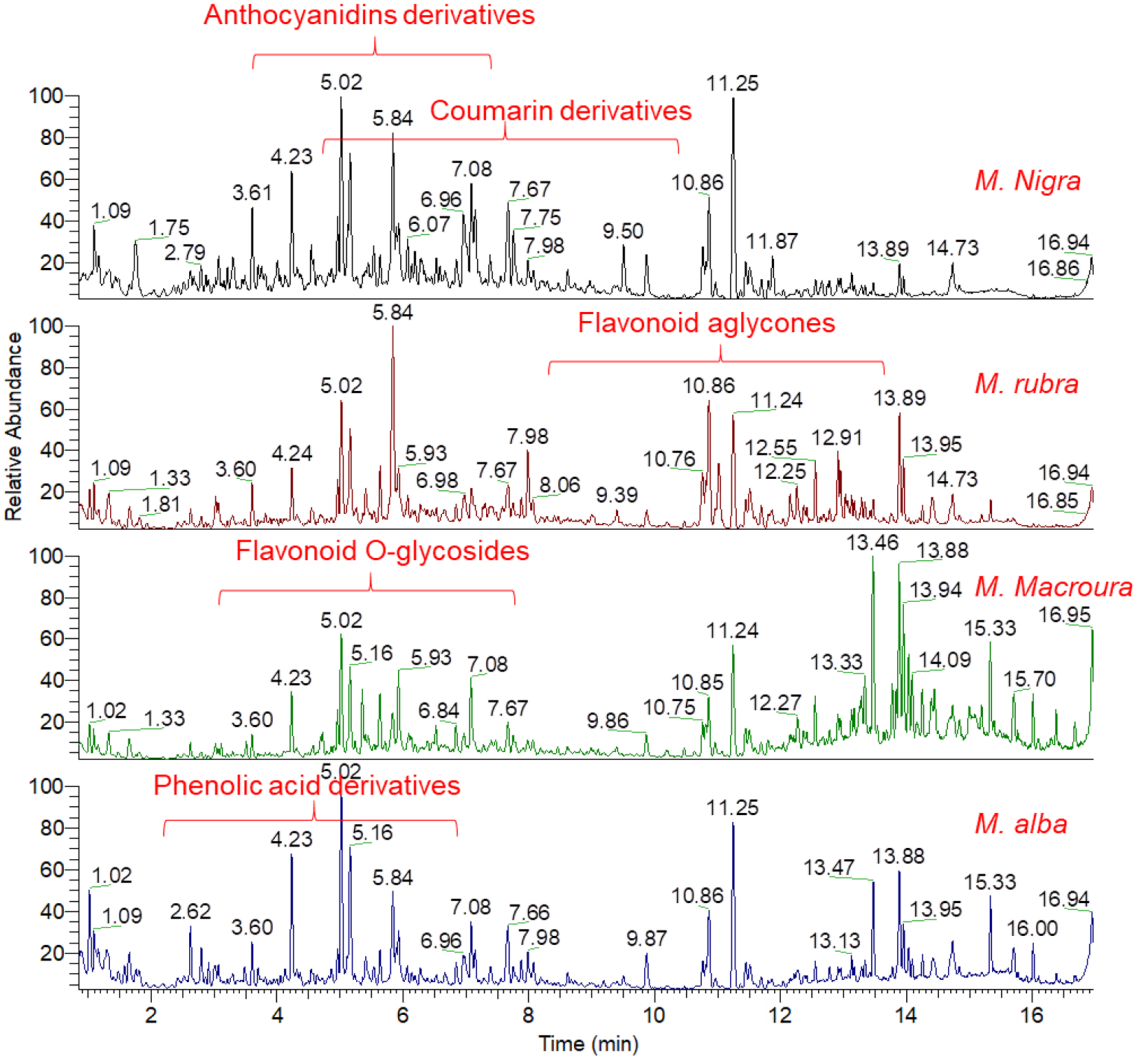
Total ion chromatogram (TIC) of the polyphenol-rich extracts from different Morus leaves analyzed by UPLC–HR–ESI– MS/MS in negative ionization mode.

This study aimed the metabolite profiling of four polyphenol-rich Morus leaves extracts (*Morus nigra*, *M*. *rubra*, *M*. *macroura* and *M*. *alba*) by ultra-performance liquid chromatography coupled to high resolution-electrospray ionization tandem mass spectrometry (UPLC-ESI-MS/MS). To better annotate compounds from their preferential ionization mode, samples were analyzed in positive and negative modes **(**Fig. 1 and Fig. S1). Identification of metabolites was achieved via comparison of retention times, quasi-molecular ion, and MS/MS fragmentation pattern to an in-house database, public databases, as well as available Morus literature^32^. A total of 61 polyphenolic compounds were tentatively identified from the four tested extracts. Compound classes included flavonoid derivatives, anthocyanidins derivatives, phenolic acids, coumarin derivatives, as well as other miscellaneous polyphenols. Table 1 summarizes the list of identified compounds, their compound classes, their structural information, and the level of abundance in different samples. An exemplary description for studying the chromatographic behavior, the molecular ion, and fragmentation pattern for annotation of some metabolites is described below in details.

**Table 1:** Annotation of metabolites from the polyphenol-rich extracts of different Morus leaves as analyzed by UPLC–HR–ESI–MS/MS in positive and negative ionization mode.

Compound Name	Formula	RT (min)	( +) ESI	Detected m/z	Delta (ppm)	(-) ESI	Detected m/z	Delta (ppm)	*M. nigra*	*M. rubra*	*M. macroura*	*M. alba*
**Flavonoid derivatives**												
Isoquercitrin	C21H20O12	5.118	(M + H) +	465.10208	− 1.44	(M-H)-	463.08884	1.38	+ +	+	+	+
Naringenin glucoside	C21H22O10	7.821	(M + H) +	435.12802	− 1.27	(M-H)-	433.11392	− 0.23	+ + +	+	+	+
Quercetin diglucoside	C27H30O17	5.12	(M + H) +	627.15503	− 0.87	(M-H)-	625.14117	0.24	+ +	+	+	+
Kaempferol diglucoside	C27H30O16	5.53	(M + H) +	611.16016	− 0.83	(M-H)-	609.14661	0.82	+ +	+ +	+	+
Kaempferol methyl ether glucuronide	C22H20O12	5.64	(M + H) +	477.10202	− 1.53	(M-H)-	475.08826	0.12	+	+	+ +	+
Quercetin 3-rhamninoside	C33H40O20	6.19	(M + H) +	757.21814	− 0.57	(M-H)-	755.20502	1.32	+ + +	+	+	+
Quercetin galactosyl-rhamnoside	C27H30O16	6.53	(M + H) +	611.16052	− 0.23	(M-H)-	609.14642	0.52	+ +	+	+ +	+
Kaempferol galactosyl dirhamnoside	C33H40O19	6.59	(M + H) +	741.22314	− 0.69	(M-H)-	739.21033	1.66	+ + +	+	+	+
Quercetin rutinoside (Rutin)	C27H30O16	6.84	(M + H) +	611.16034	− 0.53	(M-H)-	609.14648	0.62	+ +	+	+ +	+
Kaempferol rhamnosylglucoside	C27H30O15	6.97	(M + H) +	595.16492	− 1.39	(M-H)-	593.15125	0.09	+ +	+	+	+
Quercetin glucoside	C21H20O12	6.97	(M + H) +	465.10214	− 1.31	(M-H)-	463.08801	− 0.4	+ +	+	+ +	+
Luteolin glucoside	C21H20O11	7.407	(M + H) +	449.10718	− 1.47	(M-H)-	447.09332	0.09	+ +	+ +	+	+
Kaempferol glucoside	C21H20O11	7.77	(M + H) +	449.10724	− 1.33	(M-H)-	447.0932	− 0.19	+ +	+	+	+
Kaempferol malonylglucoside	C24H22O14	8.07	(M + H) +	535.10803	− 0.37	(M-H)-	533.09351	− 0.32	+ +	+	+	+ +
Tetrahydroxyflavone	C15H10O6	7.63	(M + H) +	287.05441	− 2.1	(M-H)-	285.03976	− 2.44	+ +	+	+	+
Luteolin	C15H10O6	7.792	(M + H) +	287.05441	− 2.1	(M-H)-	285.03976	− 2.44	+ +	+	+	+ +
Tetrahydroxyflavone isomer	C15H10O6	8.08	(M + H) +	287.05417	− 2.95	(M-H)-	285.04062	0.55	+ +	+	+	+ +
Apigenin	C16H14O4	11.25	(M + H) +	271.09625	− 0.86	(M-H)-	-	–	+	+	+ +	+ +
Kushenol G	C25H28O8	3.63	(M + H) +	457.18445	− 2.73	(M-H)-	–	–	–	+ +	+ +	+ +
Quercetin	C15H10O7	9.51	(M + H) +	303.04929	− 2.12	(M-H)-	301.03506	− 1.03	+ + +	–	+	+ +
Kaempferol	C15H10O6	10.78	(M + H) +	287.05463	− 1.35	(M-H)-	285.04025	− 0.73	+ + +	–	+	+ +
Kushenol A	C25H28O5	7.99	(M + H) +	–	–	(M-H)-	407.18393	− 6.06	+ +	+ +	+	+
Gericudranins A	C29H24O9	3.7	(M + H) +	517.15509	11.18	(M-H)-	–	–	+ +	+	+	+ +
Kuwanon C (Mulberrin)	C25H26O6	13.519	(M + H) +	423.17938	− 1.97	(M-H)-	421.16562	− 0.1	+	+ +	+	+ +
Kuwanon L	C35H30O11	8.62	(M + H) +	–	−	(M-H)-	625.24982	− 0.58	+ +	+	+	+
Moracin N	C19H18O4	2.9	(M + H) +	311.12326	− 14.54	(M-H)-	309.11197	− 4.09	+	+ +	+	+
Kushenol D	C27H32O6	4.71	(M + H) +	–	−	(M-H)-	451.21838	12.79	+ +	+	+ +	+
**Anthocyanidins derivatives**												
6-Hydroxycyanidin	C15H10O7	5.12	(M + H) +	303.04901	− 3.03	(M-H)-	–	− 1.14	+ +	+	+	+
6-Hydroxycyanidin isomer 1	C15H10O7	5.84	(M + H) +	303.04907	− 2.83	(M-H)-	–	–	+ +	+	+ +	+
6-Hydroxycyanidin isomer 2	C15H10O7	6.16	(M + H) +	303.04929	− 2.12	(M-H)-	–	− 1.44	+ +	+	+ +	+
6-Hydroxycyanidin isomer 3	C15H10O7	7	(M + H) +	303.04929	− 2.12	(M-H)-	–	− 2.05	+ +	+	+ +	+
Delphinidin malonylglucoside	C24H22O15	7.39	(M + H) +	551.1026	− 0.99	(M-H)-	–	− 0.61	+ +	+	+	+ +
Delphinidin galactoside	C21H21O12 +	4.669	(M + H) +	465.10208	− 1.44	(M-H)-	465.10364	− 0.45	+ +	+ +	+	+
Cyanidin di-O-glucoside	C27H31O16 +	5.529	(M + H) +	611.16016	− 0.83	(M-H)-	611.16241	1.07	+ +	+	+	+
Delphinidin sophoroside	C27H31O17 +	6.14	(M + H) +	627.15491	− 1.07	(M-H)-	627.15704	0.59	+ +	+	+ +	+
Delphinidin rhamnosyl glucopyranoside	C27H31O16 +	6.528	(M + H) +	611.16034	− 0.53	(M-H)-	611.16199	0.38	+ +	+	+ +	+
Cyanidin rhamnoside	C21H21O10 +	6.587	(M + H) +	433.11261	− 0.72	(M-H)-	433.11377	− 0.58	+ +	+ +	+	–
Cyanidin sambubioside	C26H29O15 +	7.671	(M + H) +	581.14923	− 1.49	(M-H)-	–	–	+ + +	-	+	+
**Phenolic acids**												
Caffeic acid	C9H8O4	3.7	(M + H) +	181.04912	− 2.3	(M-H)-	179.03415	− 4.65	+ +	+	+	+ +
Chlorogenic acid	C16H18O9	4.24	(M + H) +	355.10202	− 0.95	(M-H)-	353.08755	− 0.71	+ +	+	+	+ +
Cryptochlorogenic acid	C16H18O9	5.02	(M + H) +	355.1019	− 1.3	(M-H)-	353.08759	− 0.62	+	+	+ +	+ +
Caffeoylquinic acid	C16H18O9	5.17	(M + H) +	355.1019	− 1.3	(M-H)-	353.08759	− 0.62	+	+	+ +	+ +
p-Coumaroylquinic acid	C16H18O8	5.64	(M + H) +	339.10672	− 2.14	(M-H)-	337.09268	− 0.62	+	+	+ +	+
Dimethyl caffeic acid	C11H12O4	9.87	(M + H) +	209.08051	− 1.58	(M-H)-	207.06554	− 3.6	+ +	+	+	+
Rosmarinic acid	C18H16O8	9.866	(M + H) +	361.09122	− 1.6	(M-H)-	359.07733	0.25	+ +	+	+	+
**Phenolic acid derivatives**												
Dihydroferulic acid glucuronide	C16H20O10	2.86	(M + H) +	373.11246	− 1.25	(M-H)-	371.0983	− 0.2	+ +	+ +	+	+
Ginnalin B	C13H16O9	3.6	(M + H) +	317.08597	− 2.34	(M-H)-	315.07199	− 0.52	+	+ +	+	+ +
Sinapoyl glucose	C17H22O10	5.4	(M + H) +	387.12808	− 1.27	(M-H)-	385.11401	− 0.02	+ +	+	+	+ +
Isosalicin	C13H18O7	3.03	(M + H) +	287.10971	− 9.82	(M-H)-	285.09775	− 0.8	–-	+ +	+ +	+ +
**Phenolic glycosides**												
Phlorin	C12H16O8	1.64	(M + H) +	289.09128	− 1.78	(M-H)-	287.0773	0.21	+	+ +	+	+ +
Salicylic acid-hexoside	C13H16O8	4.72	(M + H) +	301.09082	− 3.23	(M-H)-	299.07715	− 0.31	-	+ +	+ +	+
Vanilloloside	C14H20O8	5.41	(M + H) +	317.12253	− 1.78	(M-H)-	315.10843	− 0.35	+ +	+ +	+ +	+
**Coumarin derivatives**												
4,7-DiHydroxy coumarin	C9H6O4	4.57	(M + H) +	179.03362	− 1.51	(M-H)-	–	− 4.74	+ +	+ +	+	+
Dihydroxycoumarin hexoside (Esculin)	C15H16O9	4.58	(M + H) +	341.08618	− 1.54	(M-H)-	–	− 0.66	+ +	+ +	+	+
4,7-Dihydroxy coumarin iosmer	C9H6O4	5.41	(M + H) +	179.03371	− 1	(M-H)-	–	− 4.91	+ +	+	+	+ +
3-Hydroxycoumarin	C9H6O3	9.87	(M + H) +	163.0387	− 1.68	(M-H)-	–	–	+ +	+	+	+
Gravelliferone	C19H22O3	14.72	(M + H) +	299.15976	− 14.75	(M-H)-	–	–	+ +	+ +	+ +	+
**Miscellaneous polyphenols**												
Reseveratrol	C12H20O4	11.25	(M + H) +	229.14314	− 1.28	(M-H)-	227.12828	− 2.65	+ +	+	+	+
Syringaresinol glucoside	C28H36O13	7.76	(M + H) +	581.22174	− 1.94	(M-H)-	579.20862	0.52	+ +	+	+	+
Guaiacylglycerol glucoside	C16H24O10	2.79	(M + H) +	377.14154	− 7.11	(M-H)-	375.12955	− 0.33	+ +	+	+	+ +
Kuwanon V	C40H38O8	14.41	(M + H) +	647.27026	9.76	(M-H)-	–	–	+	+ +	+ +	+

**Figure 2:**
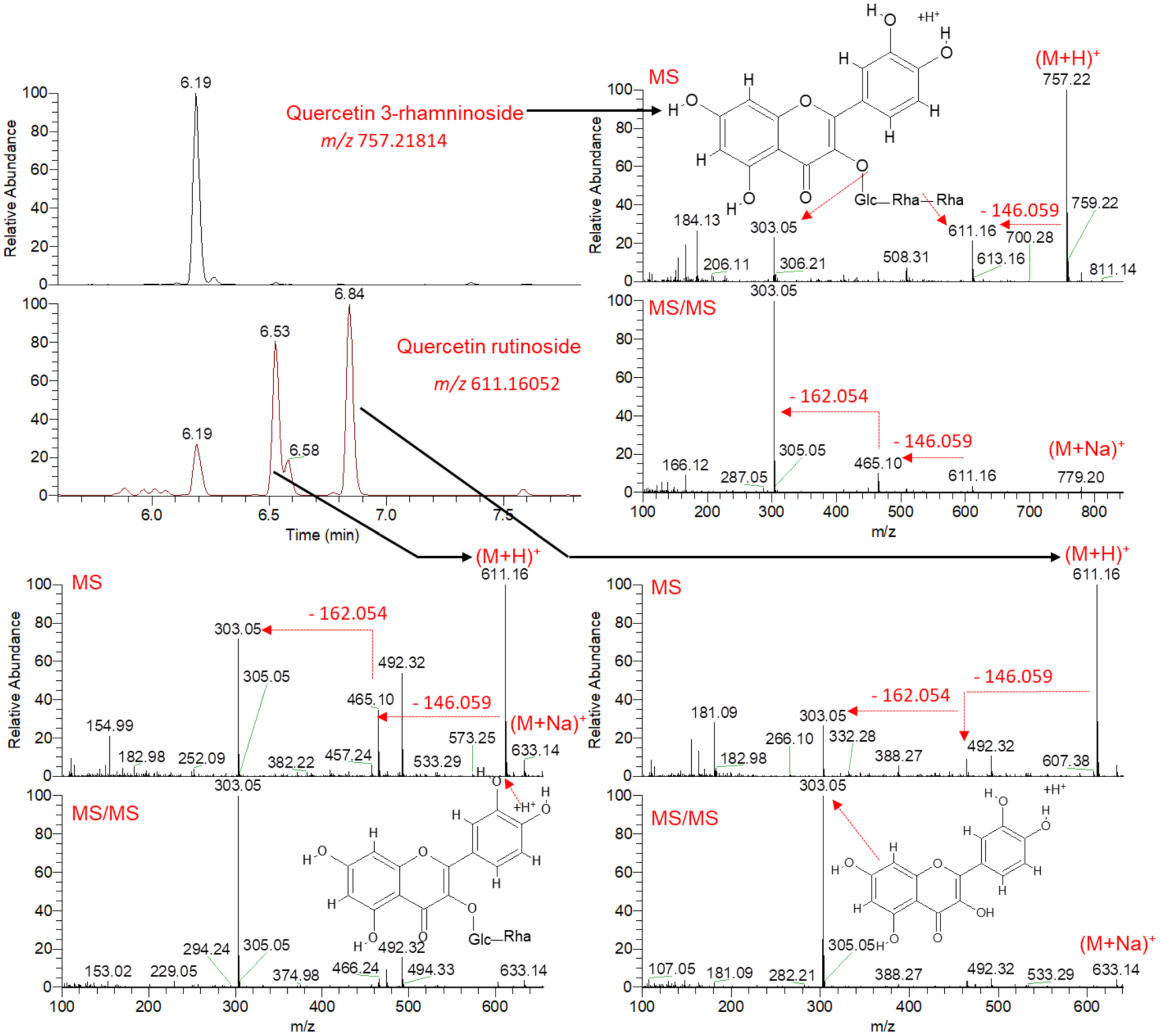
Annotation of quercetin glycosides of the polyphenol-rich extracts from different Morus leaves as analyzed by UPLC–HR–ESI–MS/MS in positive ionization mode.

Flavonoid aglycones as well as their glycosides constitute a major portion of the Morus metabolome^32^. An extracted ion chromatogram (EIC) for the ion observed at *m/z* 611.16052 showed three prominent peaks at 6.19, 6.53 and 6.84 min in positive ionization mode **(**Fig. 2**)**. The same peaks were also detected at similar retention times when the peak at *m/z* 609.14648 was extracted in negative ionization mode (Fig. S2). The MS spectrum showed that two peaks can be assigned at RT 6.53 and 6.84 min to *m/z* 611.16052 and 609.14648 as molecular ion peaks for protonated (M + H)^+^ and deprotonated (M-H)^-^ adducts, respectively. The peak at RT 6.19 min indicated that the ions detected at *m/z* 611.16052 and 609.14648 are fragments of the molecular ion at *m/z* 757.21814 (M + H)^+^ and 755.20502 (M-H)^−^. The chemical formulae assigned for both compounds were C_33_H_40_O_20_ for the exact mass of 756.211 and C_27_H_30_O_16_ for the exact mass of 610.153. The MS/MS spectra showed that the fragmentation pattern of these compounds produced a common ion at *m/z* 303.05 and 300.03, similar to quercetin in positive and negative ionization modes, respectively. This fragment is produced for neutral losses of sugars (−146.059 and −162.054 for the neutral loss of deoxy hexose and hexose moiety, respectively). The compound detected at RT 6.19 min and *m/z* 757.21814 (M + H)^+^ was firstly fragmented through a neutral loss of a deoxy hexose (e.g., rhamnose, −146.059) producing an ion at *m/z* 611.16. Further, this ion is fragmented in by a neutral loss of a second deoxy hexose (e.g., rhamnose, −146.059) as well as a hexose moiety (e.g. glucose, −162.054) producing the aglycone at *m/z* 303.05 as a base peak ion, characteristic to a protonated quercetin (M + H)^+^. This distinctive fragmentation pattern was also detected in negative ionization mode. The MS and MS/MS characteristics of this compound corresponded to quercetin *O*-rhamnosyl-*O*-rhamnosyl-*O*-hexoside (e.g., quercetin 3-rhamninoside)^32,33^. The isomers detected at RT 6.53 and 6.84 min and *m/z* 611.16052 (M + H)^+^ were firstly fragmented through a neutral loss of a deoxy hexose (e.g., rhamnose, −146.059) producing an ion at *m/z* 465.10. Moreover, this ion is fragmented by a neutral loss of a hexose moiety (e.g., glucose, −162.054), producing the aglycone at *m/z* 303.05 as a base peak ion, characteristic to protonated quercetin (M + H)^+^. This distictive fragmentation pattern was also detected in negative ionization mode. The MS and MS/MS characteristics of this compound matched quercetin *O*-rhamnosyl-*O*-glucoside (e.g., quercetin 3-*O*-rutinoside, rutin).

**Figure 3:**
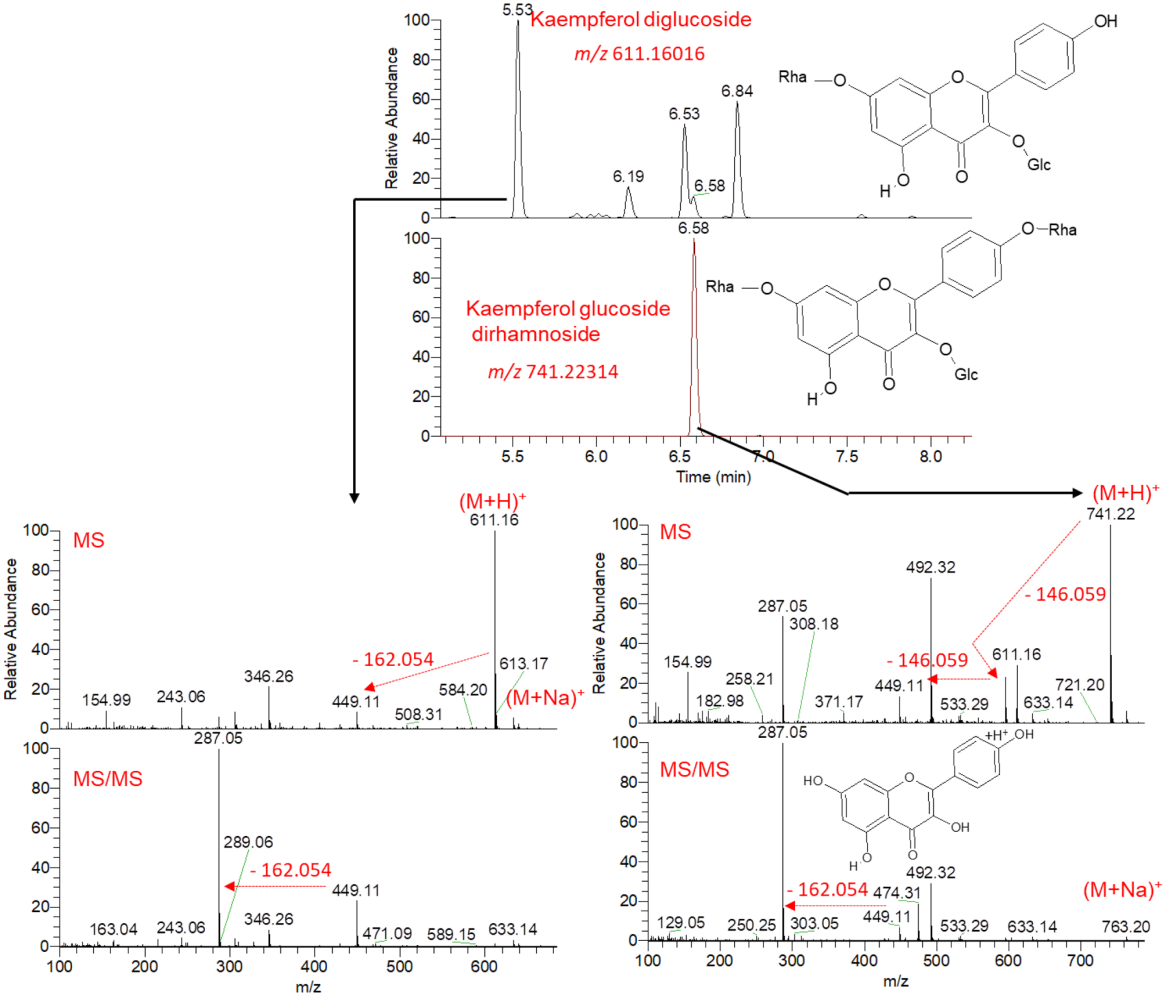
Annotation of kaempferol glycosides of the polyphenol-rich extracts from different Morus leaves as analyzed by UPLC–HR–ESI–MS/MS in positive ionization mode.

Another example is describing an extracted ion chromatogram (EIC) for the ions observed at *m/z* 611.16016 and *m/z* 741.22314, showing two prominent peaks at 5.53 and 6.58 min in positive ionization mode, respectively (Fig. 3). The same peaks were also detected at similar retention time when the peaks at *m/z* 609.14661 and 739.21033 were extracted in negative ionization mode (Fig. S3). The MS spectrum showed that peaks can be assigned to protonated (M + H)^+^ and deprotonated (M-H)^−^ adducts in their respective ionization mode. The chemical formulae assigned for both compounds were C_27_H_30_O_16_ for the exact mass of 610.153 and C_33_H_40_O_19_ for the exact mass of 740.216. The MS/MS spectra showed that the fragmentation pattern of these compounds produced a common ion at *m/z* 287.05 and 285.04 comparable to kaempferol in positive and negative ionization modes, respectively. This fragment is produced for neutral losses of sugars (−146.059 and −162.054 for the neutral loss of deoxy hexose and hexose moiety, respectively). The compound detected at RT 5.53 min, and *m/z* 611.16016 (M + H)^+^ was firstly fragmented through a neutral loss of a hexose moiety (e.g., glucose, −162.054), producing an ion at *m/z* 449.11. Further, this ion is fragmented by a neutral loss of a second hexose moiety (e.g., glucose, −162.054), producing the aglycone at *m/z* 287.05 as a base peak ion, characteristic to a protonated kaempferol (M + H)^+^. This distinctive fragmentation pattern was also detected in negative ionization mode. The MS and MS/MS characteristics of this compound matched kaempferol diglucoside. The compound detected at RT 6.58 min and *m/z* 741.22314 (M + H)^+^ was firstly fragmented through a neutral loss of a deoxy hexose (e.g., rhamnose, -146.059), producing an ion at *m/z* 595.16. Further, this ion is fragmented by neutral losses of hexose and deoxy hexose moieties producing the aglycone at *m/z* 287.05 as a base peak ion, characteristic to protonated kaempferol (M + H)^+^. This characteristic fragmentation pattern was also detected in negative ionization mode. The MS and MS/MS characteristics of this compound coincided with kaempferol which is glycosylated by one hexose and two deoxy hexose moieties (e.g., kaempferol glucoside dirhamnoside).

### Multivariate analysis of data obtained from the four polyphenol-rich Morus leaves extracts

**Figure 4:**
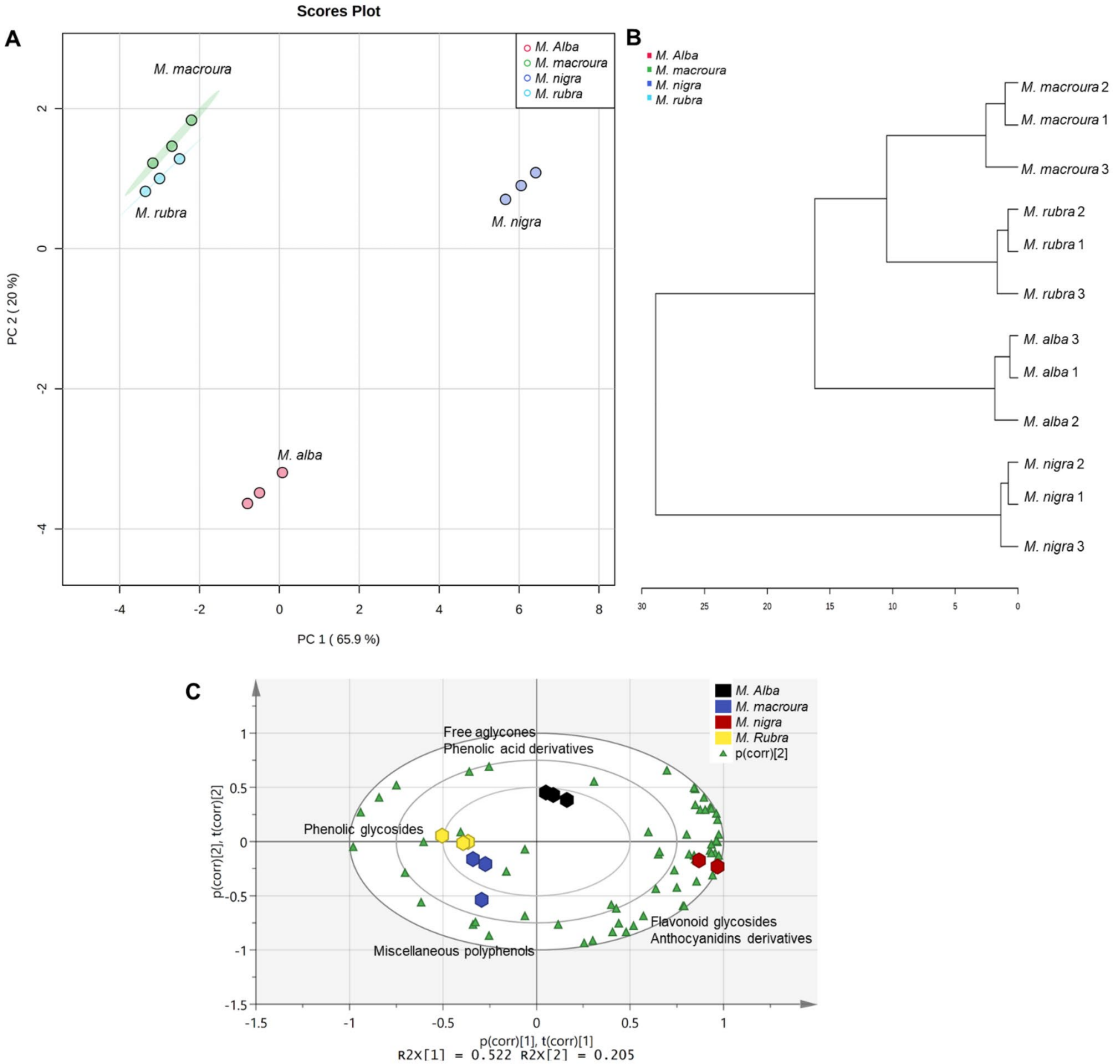
Principal component analysis (PCA) score plot (**A**), hierarchical cluster analysis (HCA) dendrogram (**B**) and PCA-biplot (**C**) based on the identified metabolites from the polyphenol-rich extracts of different Morus leaves.

An unsupervised recognition analysis was performed using principal component analysis (PCA) and hierarchical cluster analysis (HCA), showing a clear segregation of species, reflecting their diverse metabolomes (Fig. 4). *M. nigra* samples were clearly separated from other species indicating a greater variation in its metabolome. Meanwhile, in a second cluster, *M*. *alba* was clearly separated from *M*. *rubra* and *M*. *macroura,* which were separately sub-clustered. The PCA biplot allowed simultaneous display and interpretation of scores and loadings from analyzed samples. Samples are allocated near metabolites that contribute higher to samples discrimination.

**Figure 5:**
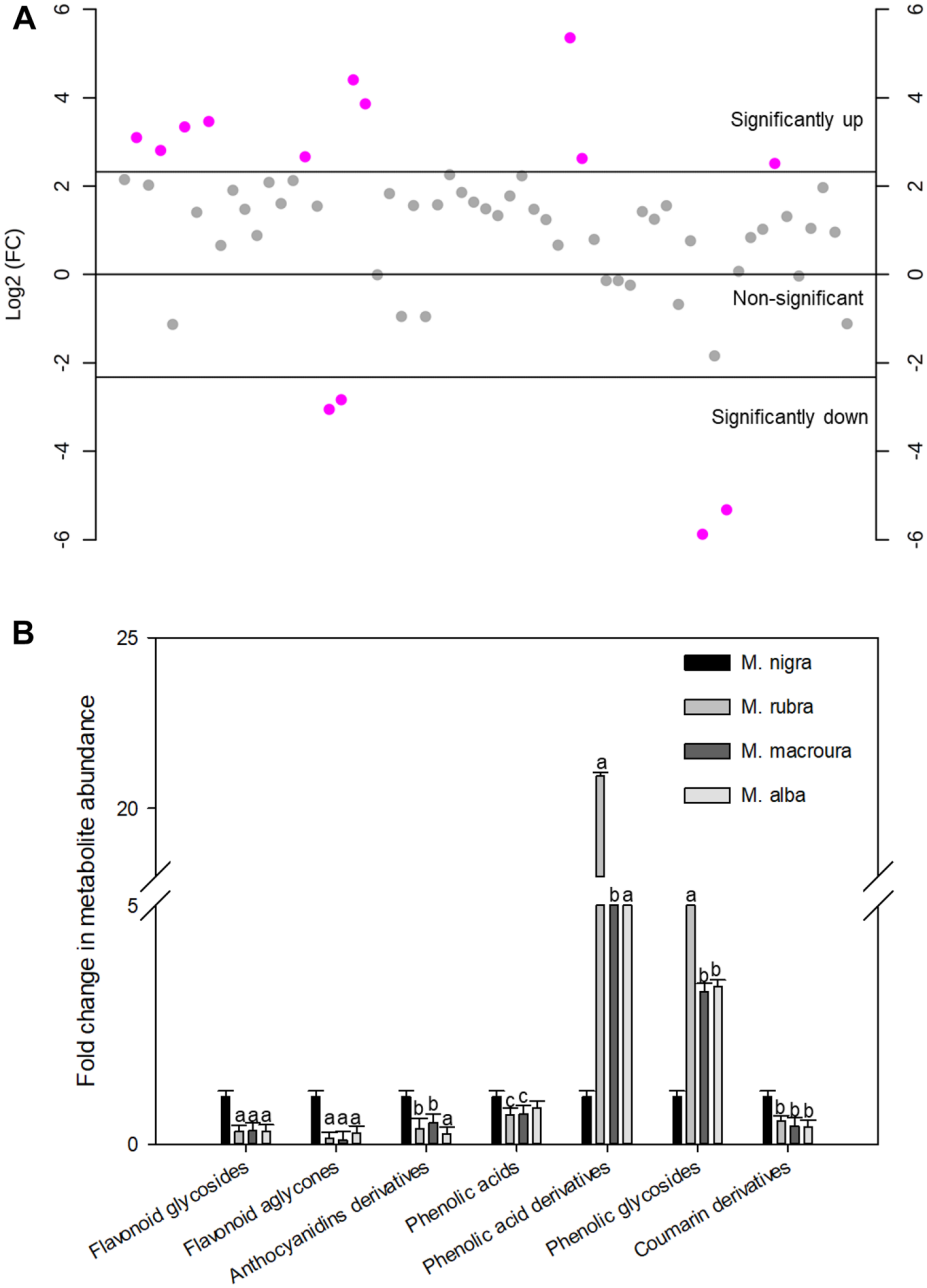
Fold change (FC) analysis of metabolites (**A**) and classes (**B**) that were significantly changed in *Morus nigra* leaves relative to other Morus species. Metabolites with FC (calculated relative to *M. nigra.*) > 5 and *p*-value < 0.05 were considered significantly changed. ^a,b,c^indicate significant changes at *p* < 0.001, 0.01 and 0.05, respectively.

**Figure 6:**
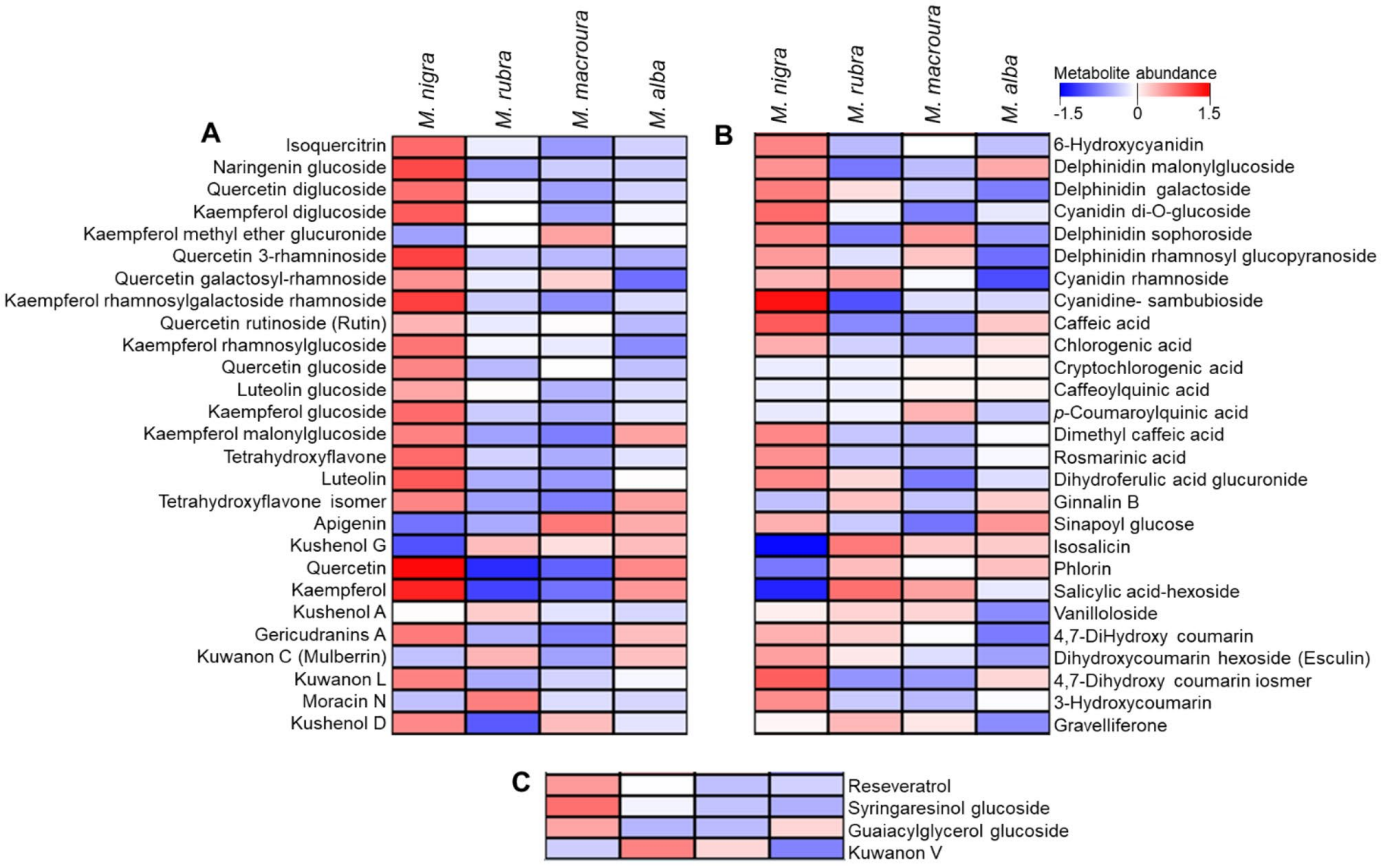
Heat map for the distribution of metabolites identified from the polyphenol-rich extracts of different Morus leaves. (**A**) represents metabolites belonging to flavonoid class, (**B**) represents metabolites belonging to anthocyanidins, phenolic acids and coumarin derivatives and (**C**) represents miscellaneous polyphenols. The color scale represents the log_10_-scaled values of metabolite abundance.

To further identify the discriminating markers for *M. nigra* from other tested polyphenol-rich Morus leaves extracts, a supervised analysis was performed using an orthogonal projection to latent structures discriminant analysis (OPLS-DA) (Fig. S4). The calculated R2Y value (the explained variance, a goodness-of-fit value) and Q2 value (the predictive capability, goodness-of-prediction value) of the OPLS-DA model were greater than 0.9, indicating model reliability. The obtained OPLS-DA model was further cross-validated using permutation analysis (100 times) to reduce the risk of overfitting. Metabolites showing fold change > 5 (relative to *M. nigra*) and *p* value < 0.05 were considered significantly changed. Fold change (FC) analysis revealed ten metabolites that are discriminatory chemical markers for *M. nigra* (Fig. 5 and Table S1). Intriguingly, *M. nigra* showed a significant accumulation of metabolites from flavonoids, anthocyanidins, phenolic acids, and coumarin derivatives. Meanwhile, *M*. *rubra* and *M*. *macroura* showed significant accumulation of phenolic acid derivatives and phenolic glycosides. Conversely, *M*. *alba* accumulated the least polyphenol content (Fig. 6).

**Figure 7:**
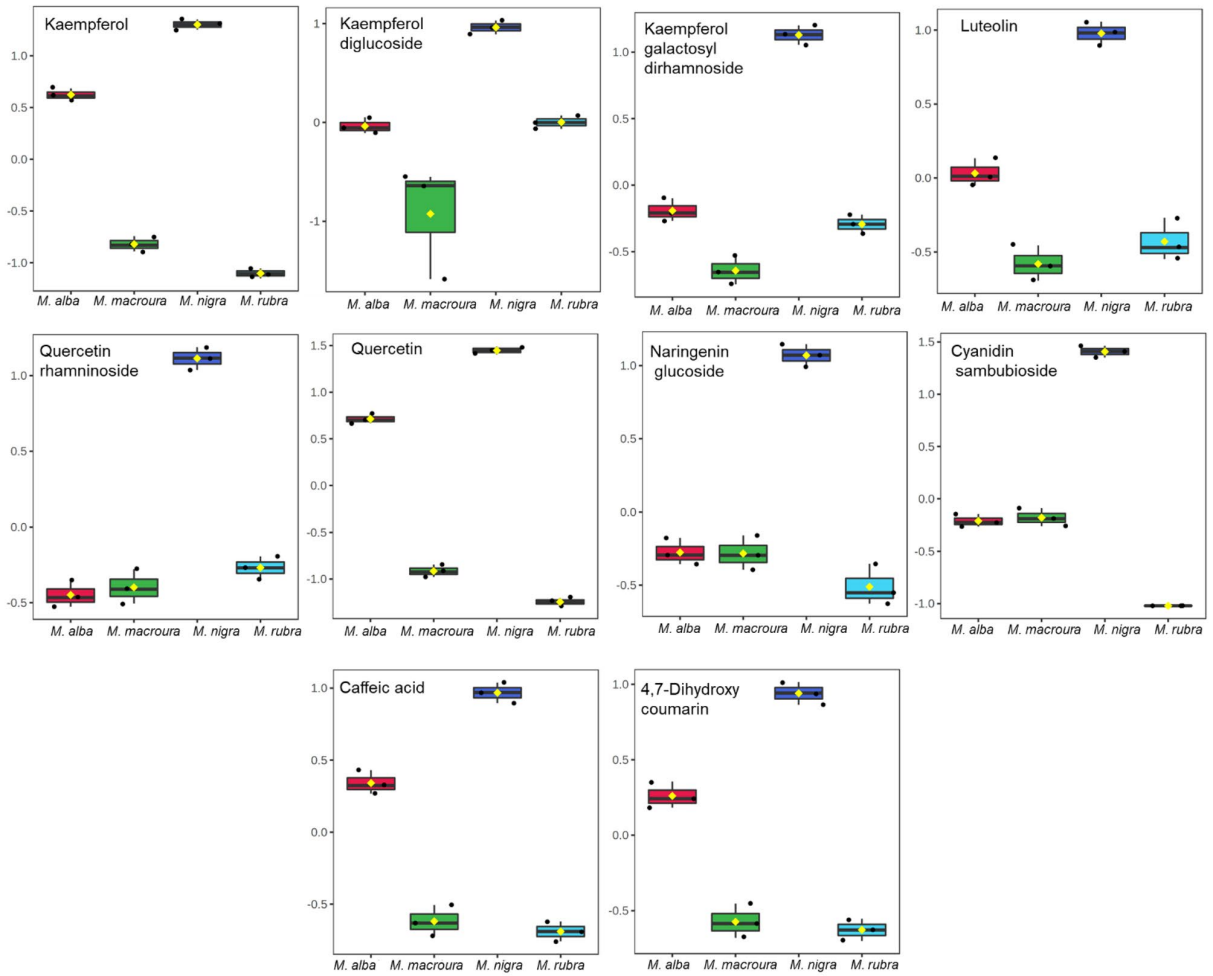
Metabolites with high abundance in the polyphenol-rich extracts from *Morus nigra* leaves. The *y*-axis represents the log_10_-scaled values of metabolite abundance.

Flavonoid aglycones (kaempferol, luteolin, and quercetin) as well as the glycosides of kaempferol, quercetin and naringenin next to an anthocyanidin derivative (cyaniding sambubioside), phenolic acid (caffeic acid) and a coumarin derivative (4,7-dihydroxy coumarin) were found to be the discriminating chemical markers of *Morus nigra* (Fig. 7). Salicylic acid hexoside and iso-salicin discriminated *M*. *rubra*, while apigenin discriminated *M*. *macroura* (Fig. S5).

### Total phenolics and flavonoids contents of Morus leaves extracts

The standard calibration curves of gallic acid and rutin were constructed (Figure S6), and the total phenolics and flavonoids contents were determined. The amount of total phenolics in the leaves extract of *M. nigra* was the highest (79.0 ± 4.7 mg gallic acid equivalent/g dry extract) compared to other leaf extracts. *M*. *rubra*, *M*. *macroura* and *M*. *alba* showed 61.83 ± 2.49, 52.4 ± 3.48 and 45.5 ± 2.3 mg gallic acid equivalent/g dry extract, respectively. Meanwhile, the total flavonoids content in the leaves extract of *M. macroura* was the highest (40.33 ± 3.29 mg rutin equivalent/g dry extract) compared to other leaf extracts. The total flavonoids for *M*. *nigra*, *M*. *rubra,* and *M*. *alba* were 28.16 ± 1.17, 9.0 ± 0.47, and 8.6 ± 0.47 mg rutin equivalent/g dry extract, respectively (Fig. S7).

### Bacterial Isolates and phenotypic identification

Gram stain was applied on the collected isolates and the microscopical examination showed Gram-positive cocci or coccobacilli arranged in pairs or short chains. The isolates showed catalase negative reaction and tolerance to 6.5% NaCl.

### Identification of enterococcal isolates by PCR assay

PCR results revealed that 88% of the isolated were *E. faecalis* (n = 22) at a band size of 942 bp, while the PCR results showed that 12% of the isolated were *E. faecium* (n = 3) at a band size of 535 bp.

### Assessment of biofilm formation

The qualitative assessment of biofilm formation by Congo red showed that 96% of the isolates could form biofilm. Crystal Violet assay classified the isolates into strong (n = 12; 48%), moderate (n = 7; 28%), weak (n = 5; 20%), and non-biofilm (n = 1; 4%). All strong biofilm-forming isolates (n = 12) were *E. faecalis*.

### Anti-biofilm assay

#### Micro-titer plate assay

**Figure 8:**
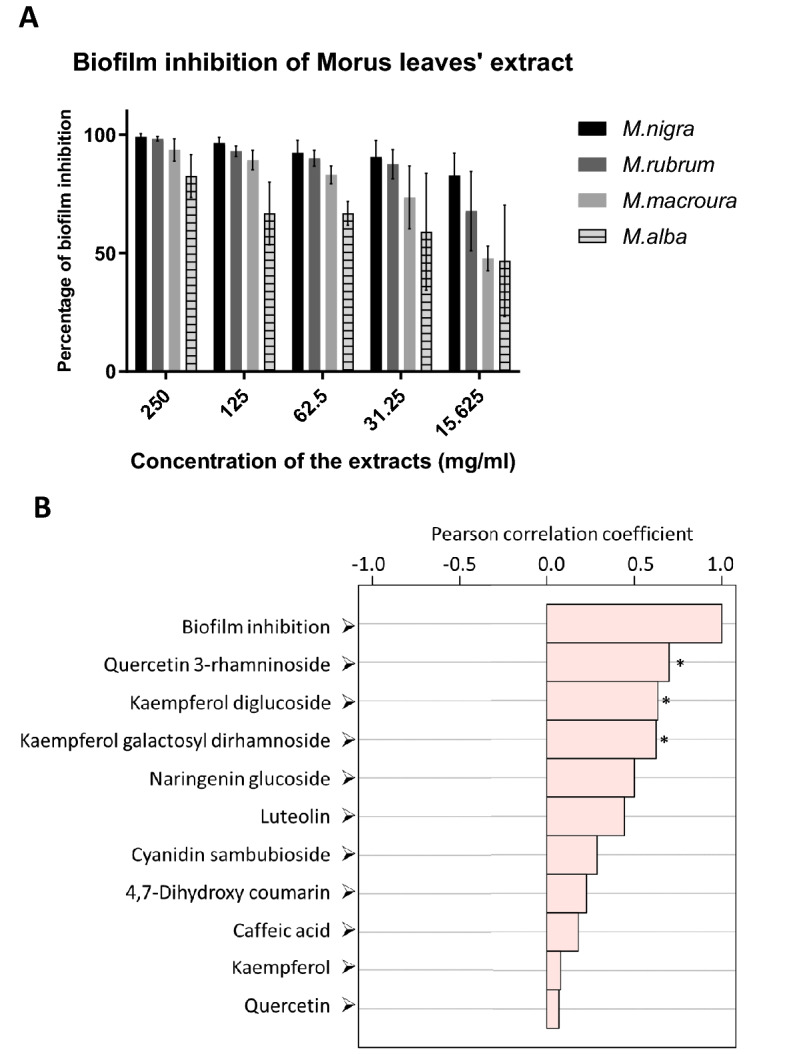
Biofilm inhibition of the four Morus leaves extract against *E. faecalis* (**A**) and top metabolites correlated with biofilm inhibitory activity (**B**). Asterisks indicate metabolites that are positively and significantly correlated (*p* < 0.05).

Inhibition of the biofilm formation by Morus leaves extract was tested on the strong biofilm- forming isolates. Results were expressed as inhibition percentages of biofilm development. At the concentration 250 mg/ml, 125 mg/ml, 62.5 mg/ml, 31.25 mg/ml and 15.625 mg/ml (Fig. 8). *M. nigra* leaves extract exerted the maximum biofilm inhibition with percentages 99 ± 1.41%, 96.5 ± 2.38%, 92 ± 5.32%, 90.5 ± 6.95% and 82.75 ± 9.43% respectively. *M. rubra* leaves extract followed *M. nigra* leaves extract with biofilm inhibition percentages 98.25 ± 0.96%, 93 ± 2.16%, 90 ± 3.37%, 87.5 ± 6.14% and 67.75 ± 16.76% at the concentrations 250 mg/ml, 125 mg/ml, 62.5 mg/ml, 31.25 mg/ml, and 15.625 mg/ml respectively. For *M. macroura* leaves extract, the inhibition of biofilm was 93.5 ± 4.73%, 89.25 ± 4.11%, 83 ± 3.74%, 74 ± 13.23%, and 47.75 ± 5.19%. The least biofilm percent inhibition was observed with *M. alba* leaves; 82.5 ± 9%, 66.75 ± 13.23%, 66.75 ± 5.06%, 59 ± 24.68% and 46.75 ± 23.54% at concentrations 250 mg/ml, 125 mg/l, 62.5 mg/ml, 31.25 mg/ml, and 15.625 mg/ml respectively. Quercetin 3-rhamninoside, kaempferol diglucoside and kaempferol galactosyl dirhamnoside were found to be positively and significantly correlated with the biofilm inhibition (Fig. 8).

## Discussion

Biofilms are microbial communities of surface-attached cells confined in extracellular polymeric substances (EPS); this is considered a natural habitat for the microbial cells’ adaptation^34,35^. Biofilms are involved in 80% of all microbial infections in the body. The bacteria in biofilm are more resistant to antimicrobial agents than their free counterparts, and this resistance can be intrinsic or acquired^36^. Urinary tract infections are among the most common nosocomial and community-acquired infections and are usually associated with biofilm formation^37^. Enterococci is emerging as a major cause of nosocomial UTIs^38^. *E. faecalis* is the predominant enterococcal species isolated from patients with UTIs followed by *E. faecium*^39^. In the current study, 88% of the clinical UTIs isolates were identified as E. *faecalis,* and 12% of the isolates were *E. faecium.* The abundance of *E. faecalis* over *E. faecium* in patients with UTIs was in agreement with other studies^37,40^. The ability of enterococci to cause and maintain infection in the urinary tract is related to biofilm formation either on indwelling devices like urinary catheters or urinary tract tissues themselves^41^. In the current study, 96% of the isolates formed biofilm. The high percentage of biofilm formation among enterococcal urinary isolates is consistent with other studies^42–44^. There is a crucial demand to explore novel and efficient cost-effective methods against bacterial biofilm formation^45^. Phytochemical compounds and total plant extracts have drawn attention for the treatment of bacterial infections; they have shown the ability to inhibit biofilm formation and the quorum sensing system, which regulates biofilm formation^46^. Mulberry fruits extracts are reputable for their antibacterial activity against some Gram-positive and Gram-negative strains^47^. The antibiofilm of *M. alba* has been studied against *Streptococcus mutans* and *Streptococcus sanguinis*^48^.

In the study, we aimed to explore the capability of polyphenol-rich Morus leaves extracts to inhibit biofilm formed by enterococcal clinical isolates. Our findings explored -for the first time- the antibiofilm inhibition *M. nigra*, *M*. *rubra*, *M*. *macroura* and *M*. *alba* polyphenol-rich leaves extracts against *E. faecalis* -the causative agent for urinary tract infection- in a dose-dependent manner. These results of biofilm inhibition are in accordance with their phenolic contents; where *M. nigra* reported the highest biofilm inhibition as well as the highest phenolic content, followed by *M. rubra* then *M. macroura,* and the least biofilm inhibition and phenolic contents were revealed by *M. alba*. Additionally, the four polyphenol-rich Morus leaves extracts were subjected to comprehensive non-targeted metabolic profiling using UPLC-ESI-MS/MS combined with chemometrics. Moreover, metabolic profiling could discriminate *M. nigra* with the accumulation of metabolites such as flavonoids, anthocyanidins, phenolic acids, and coumarin derivatives, *M*. *rubra* displayed phenolic acid derivatives and phenolic glycosides assembly. Further, *M*. *alba* accumulated the least polyphenol content. Flavonoid derivatives (kaempferol, luteolin, quercetin and naringenin), anthocyanidin derivative (cyanidin sambubioside), phenolic acid (caffeic acid), and a coumarin derivative (4,7-dihydroxy coumarin) were found to be the discriminating chemical markers of *M. nigra*.

Correlation analysis, as expressed by Pearson’s correlation coefficients, indicating the relationship between metabolites content and the biofilm inhibition revealed three major compounds that were positively and significantly correlated. The metabolites included quercetin 3-rhamninoside, kaempferol diglucoside and kaempferol galactosyl dirhamnoside. Intriguingly, quercetin has been reported to possess a variety of pharmacological activities, particularly antimicrobial activity against Gram-positive and Gram-negative bacteria as well as viruses and fungi^49,50^. It has been shown that quercetin exerts its antimicrobial activity through the disruption of the cell membrane and its permeability, nucleic acid biosynthesis, virulence factors expression, mitochondrial function, and biofilm formation^51^. Additionally, plant-derived quercetin has been recently shown to inhibit *E. faecalis* biofilm through disruption of protein translation and glycolytic pathways^52^. Moreover, the anti-biofilm activities of quercetin against other Gram-positive pathogens, such as *Staphylococcus aureus* and *S. epidermidis,* as well as Gram-negative *Pseudomonas aeruginosa* and *Salmonella* spp. were also reported^53–56^. Further, kaempferol was reported as a promising antimicrobial plant flavonoid that has potentiality to inhibit biofilm formation in *S. aureus*^57–59^. Our study suggests that Morus-derived flavonoids such as quercetin and kaempferol glycosides give further attention as a potential anti-biofilm agent against *E. faecalis*. Further studies are essential to compare the antimicrobial activity of free aglycones and glycosides of quercetin and kaempferol, as well as their combinations.

## Conclusion

In this study, we investigated the antibiofilm activities, and metabolites of Morus leaves by untargeted metabolomics combined with chemometrics. In conclusion, different species of Morus leaf extracts showed promising antibiofilm activities, in which the polyphenolic content and the antioxidant properties exerted by these extracts are involved. Identification of the discriminatory chemical markers of different Morus leaves extracts was achieved via non-targeted metabolomics combined with chemometrics. *M*. *nigra* accumulated significant amount of specialized polyphenols, to which the anti-biofilm activity can be correlated. Further investigations are requested to get insight into to the mechanism of action of the biofilm inhibition.

## Supplementary Information

Supplementary Information.

## Data Availability

All data generated or analysed during this study are included in this published article [and its supplementary information files].
