# Supplementary material for: Comparative metabolite profiling of four polyphenol rich Morus leaves extracts in relation to their antibiofilm activity against Enterococcus faecalis
Source: Sci Rep. 2022 Nov 23;12:20168. doi: 10.1038/s41598-022-24382-4 (PMC9691725; doi:10.1038/s41598-022-24382-4)
Supplement: Supplementary file 1 — Supplementary Information. [file 41598_2022_24382_MOESM1_ESM.pdf]

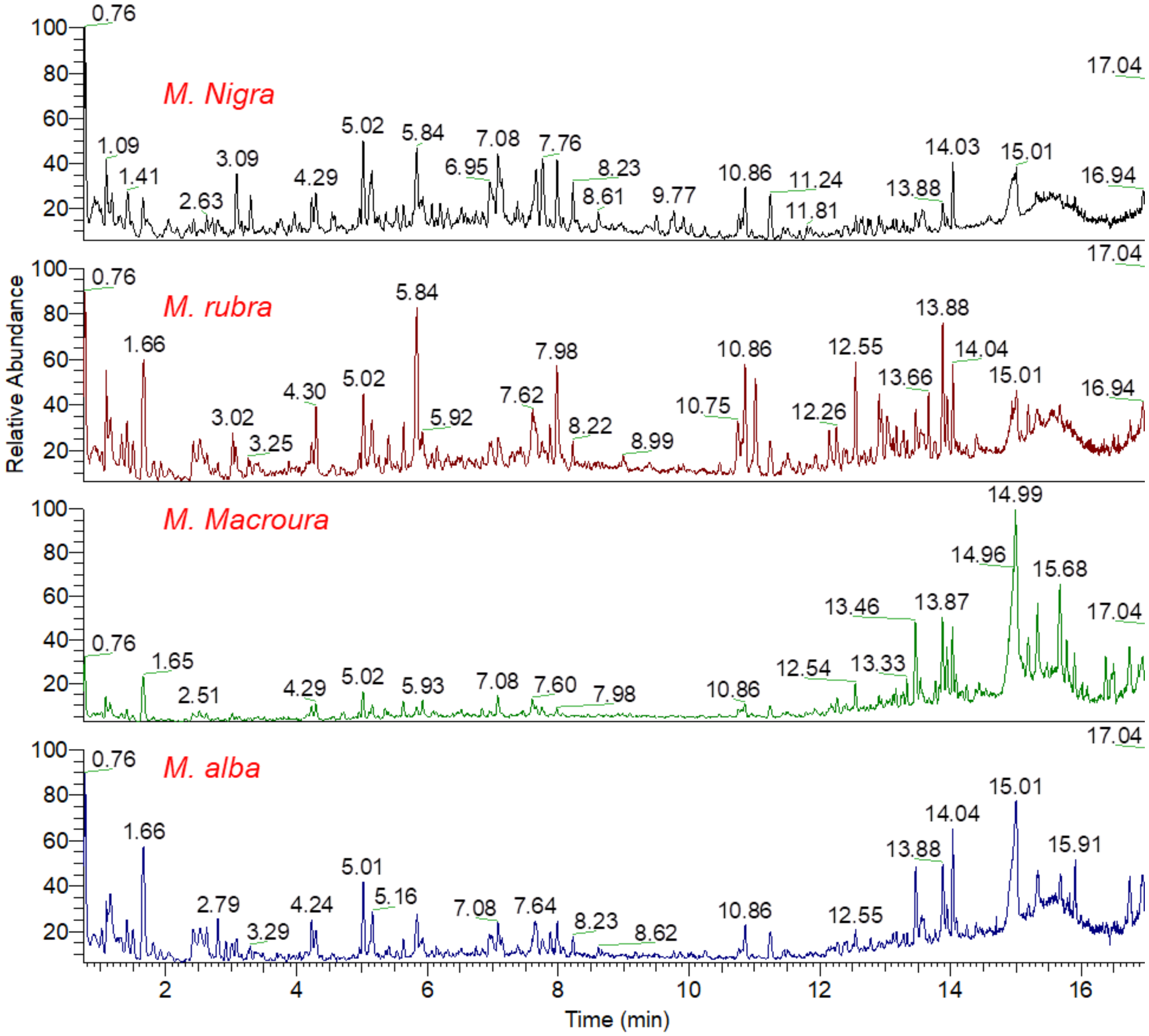

Figure S1. Total ion chromatogram (TIC) of the polyphenol-rich extracts from different Morus leaves analyzed by UPLC–HR–ESI– MS/MS in positive ionization mode.

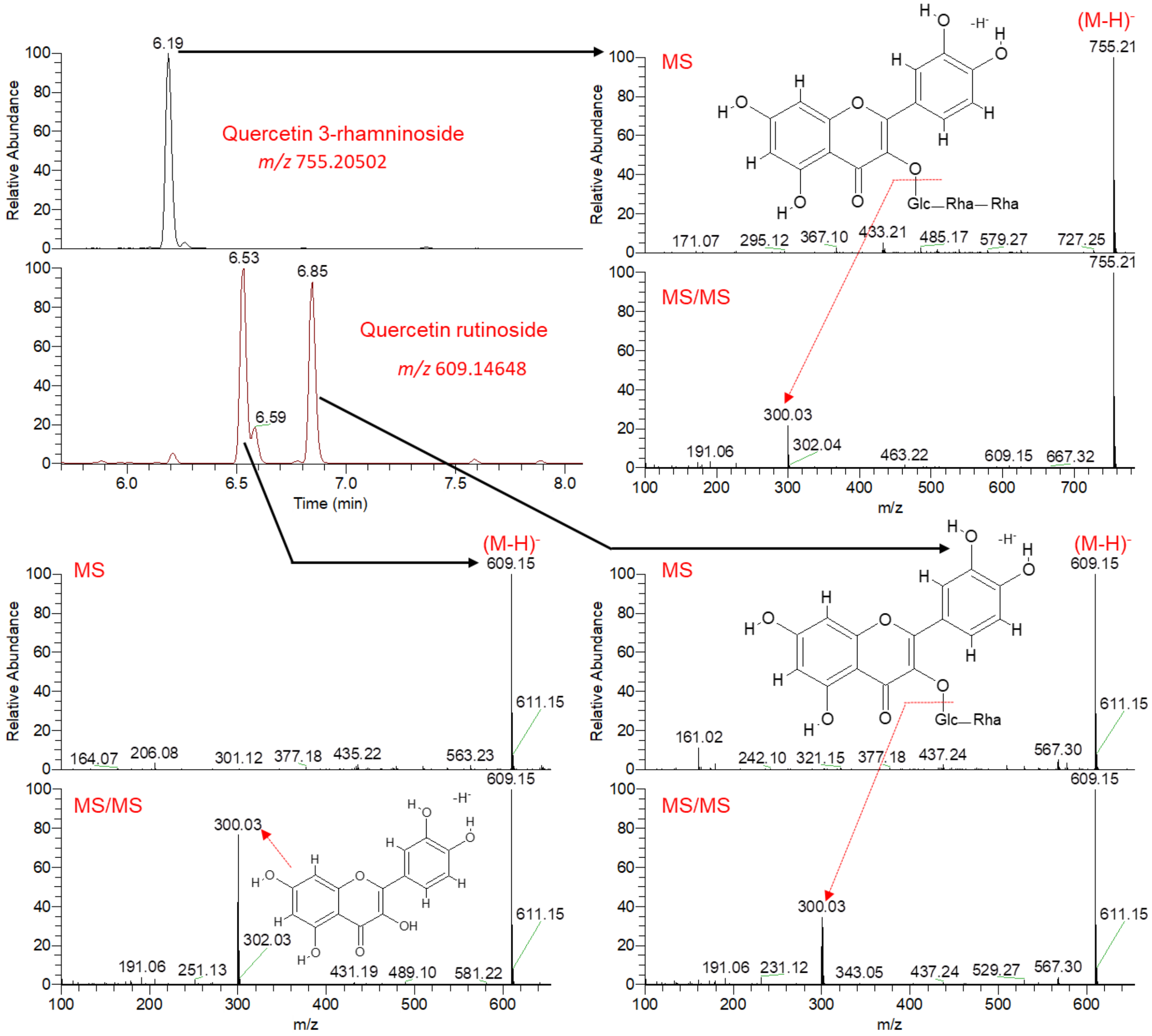

Figure S2. Annotation of quercetin glycosides of the polyphenol-rich extracts from different Morus leaves as analyzed by UPLC–HR–ESI–MS/MS in negative ionization mode.

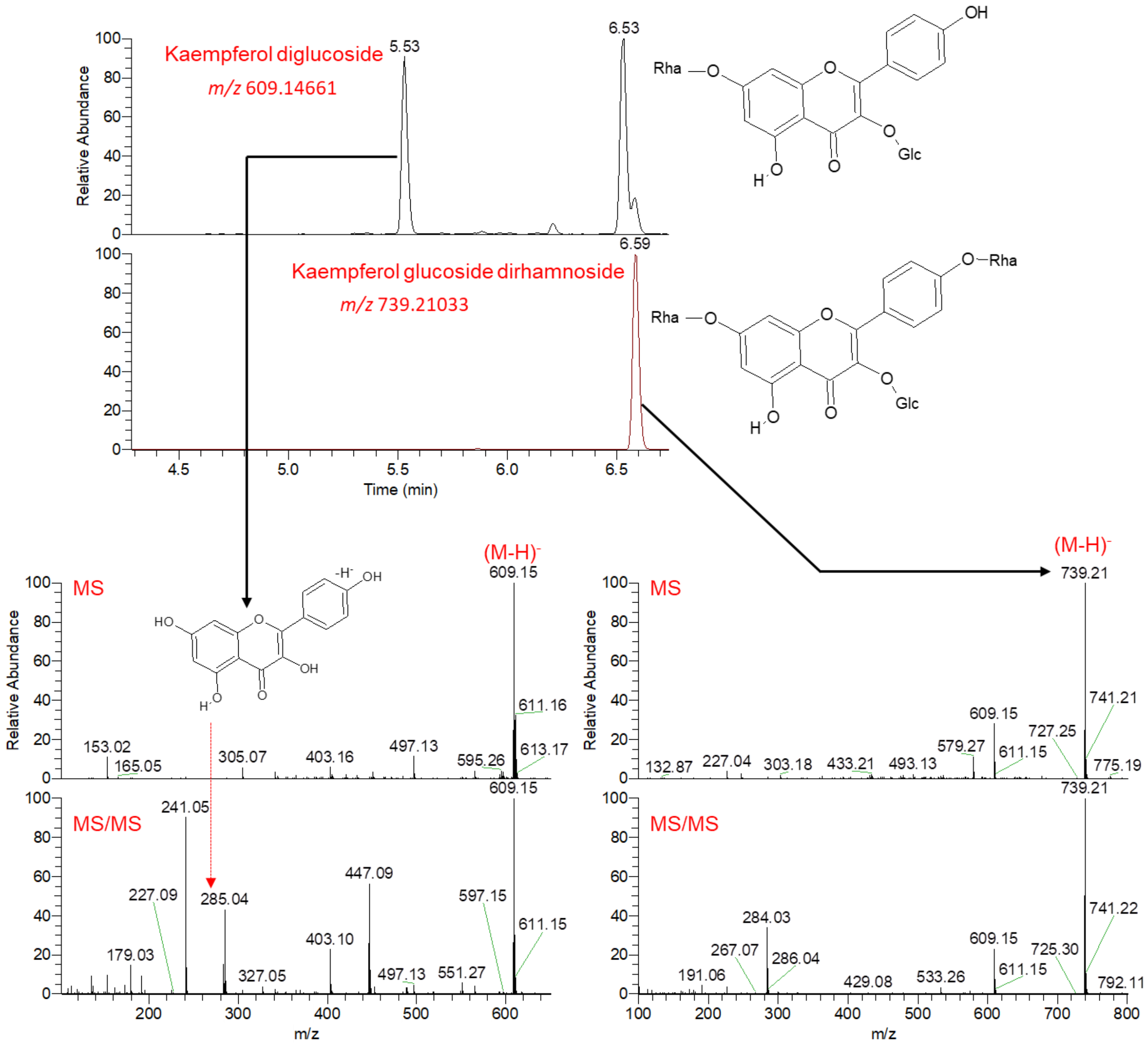

Figure S3. Annotation of kaempferol glycosides of the polyphenol-rich extracts from different Morus leaves as analyzed by UPLC–HR–ESI–MS/MS in negative ionization mode.

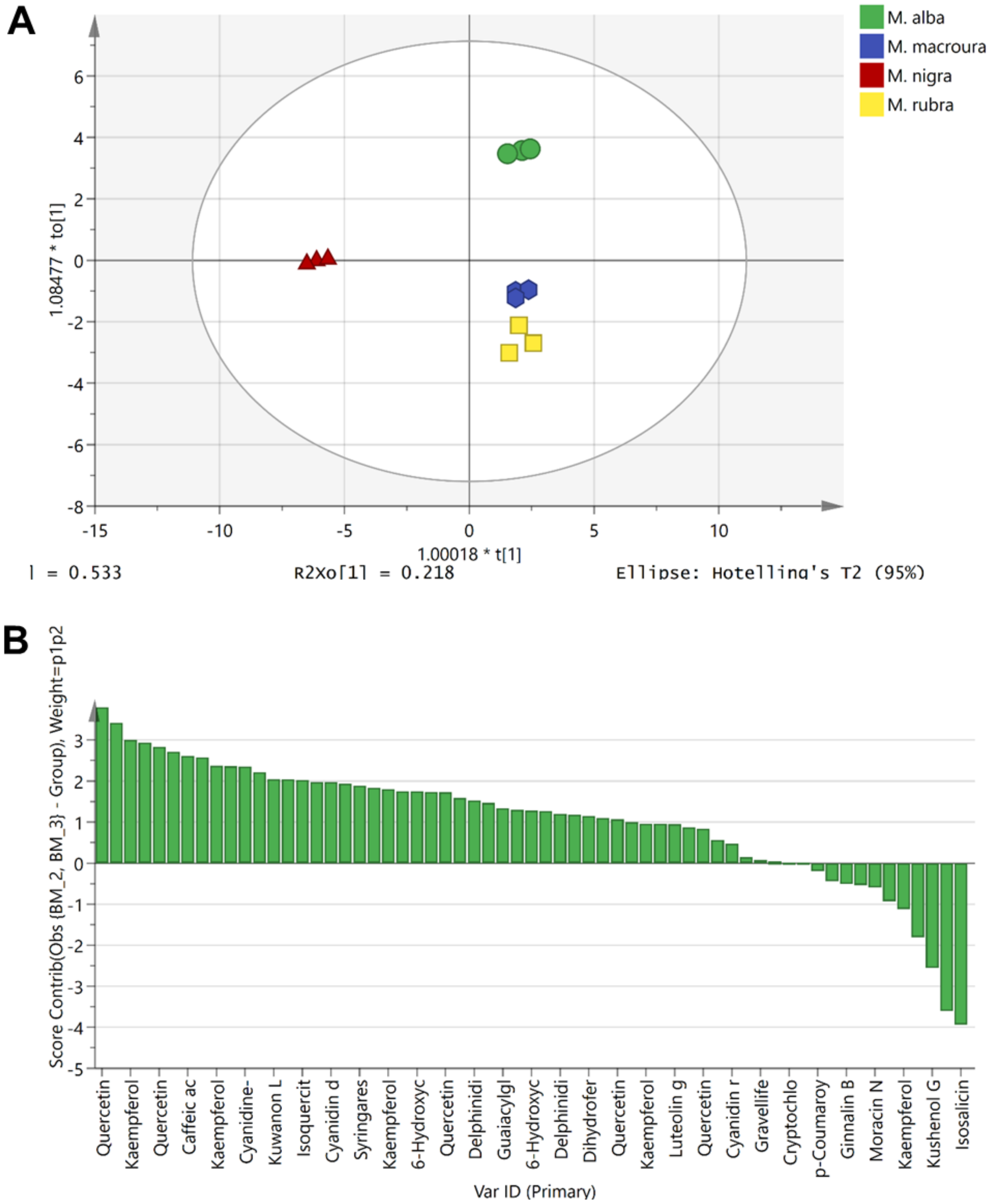

Figure S4. Orthogonal Projections to Latent Structures Discriminant Analysis (OPLS-DA) score plot (A) and Contribution plot (B) based on the identified metabolites from the polyphenol-rich extracts of different Morus leaves.

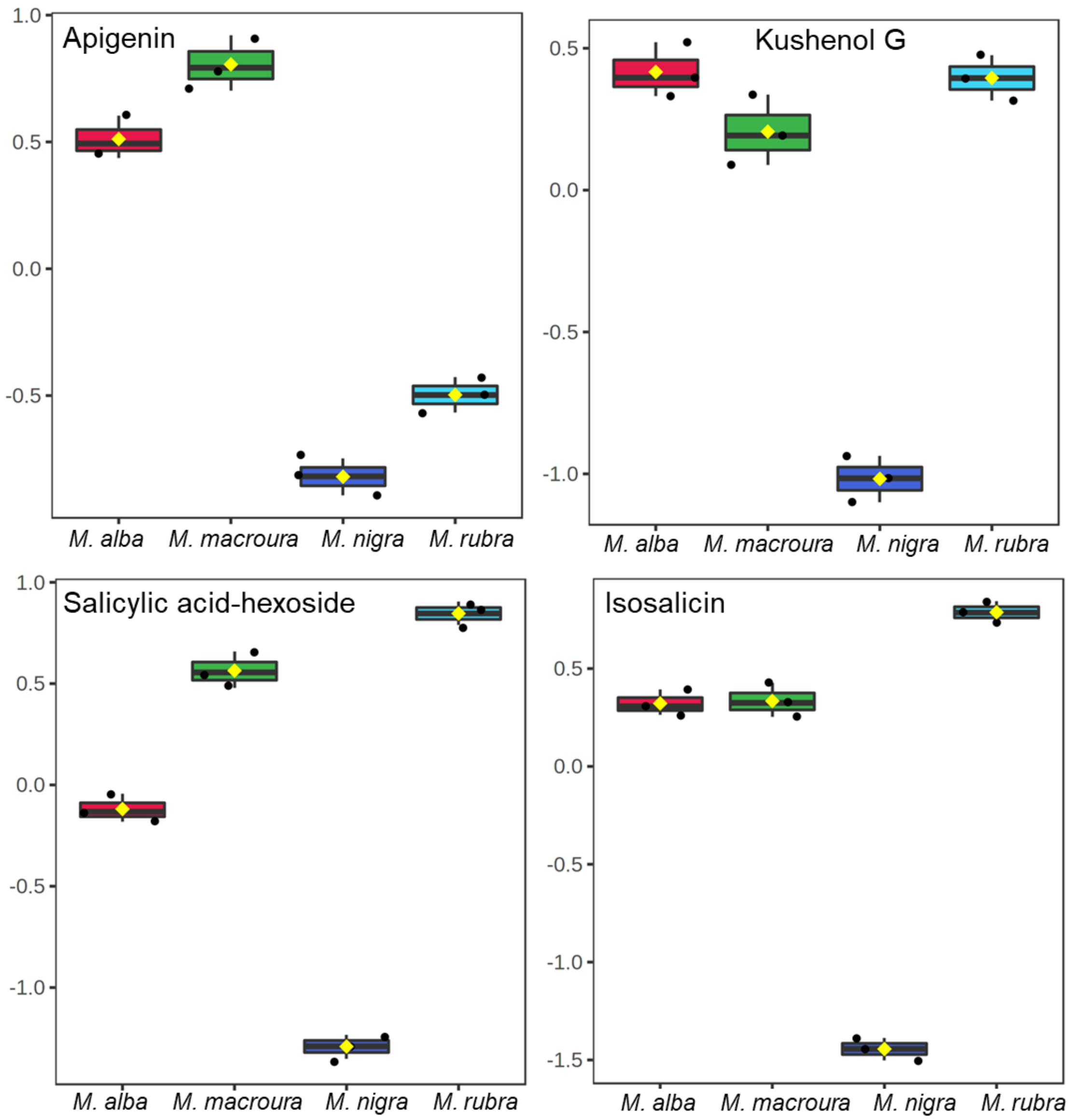

Figure S5. Metabolites with low abundance in the polyphenol-rich extracts extract from *Morus nigra* leaves. The *y*-axis represents the log_{10}-scaled values of metabolite abundance.

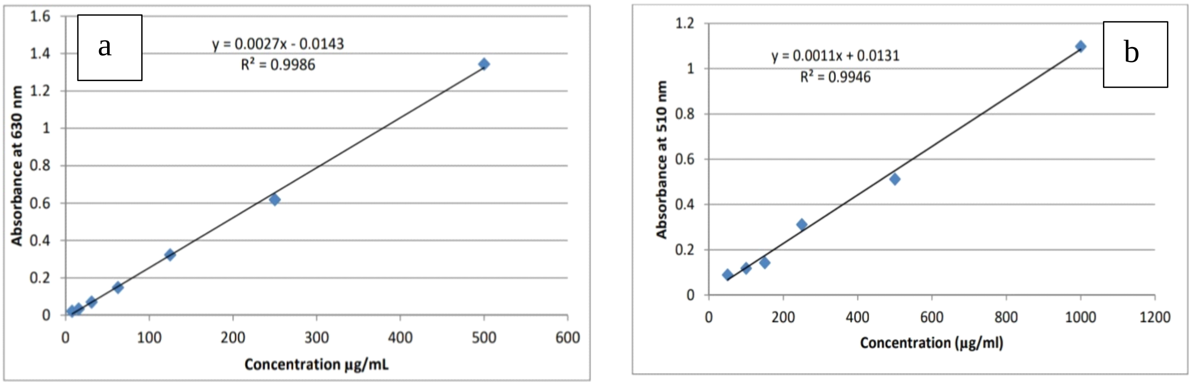

Figure S6. Representative standard curves: (a) gallic acid, (b) rutin (The average of the readings of the 6 replicates was taken)

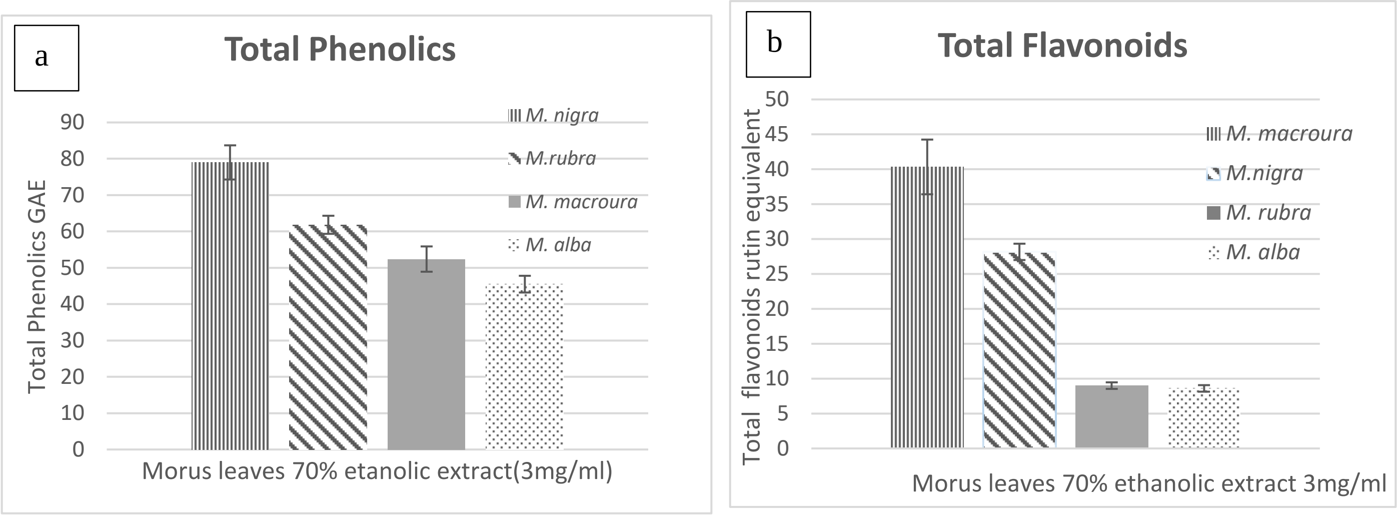

Figure S7. Total phenolics (A) and total Flavonoids (B) of the four Morus species calculated as gallic acid equivalent (GAE) and rutin equivalent, respectively.

Table S1. Fold change (FC) analysis of metabolites that were significantly changed in *Morus nigra* leaves relative to other Morus species.

Metabolites	FC	$\log_2$(FC)	*p*-value	$-\log_{10}$(p)
Cyanidin sambubioside	40.968	5.3564	2.00E-05	4.6988
Quercetin	21.184	4.4049	0.0051951	2.2844
Kaempferol	14.544	3.8623	0.004698	2.3281
Kaempferol galactosyl dirhamnoside	11.025	3.4627	4.84E-07	6.3149
Quercetin 3-rhamninoside	10.124	3.3397	1.53E-09	8.8148
Naringenin glucoside	8.5651	3.0985	3.44E-08	7.4633
Kaempferol diglucoside	7.0016	2.8077	0.0026091	2.5835
Luteolin	6.3379	2.664	2.52E-05	4.5989
Caffeic acid	6.1743	2.6263	0.0016105	2.793
4,7-Dihydroxy coumarin	5.7097	2.5134	0.00074817	3.126
Kushenol G	0.14027	-2.8338	1.57E-08	7.8033
Apigenin	0.12055	-3.0523	0.011923	1.9236
Salicylic acid-hexoside	0.024961	-5.3242	5.87E-05	4.2314
Isosalicin	0.017007	-5.8778	9.77E-08	7.0099
